# Increased Cortical Activity in Novices Compared to Experts During Table Tennis: A Whole-Brain fNIRS Study Using Threshold-Free Cluster Enhancement Analysis

**DOI:** 10.1007/s10548-023-00963-y

**Published:** 2023-04-29

**Authors:** Daniel Carius, Fabian Herold, Martina Clauß, Elisabeth Kaminski, Florian Wagemann, Clemens Sterl, Patrick Ragert

**Affiliations:** 1 Department of Movement Neuroscience, Faculty of Sport Science, Leipzig University, 04109 Leipzig, Germany; 2 Max Planck Institute for Human Cognitive and Brain Sciences, 04103 Leipzig, Germany; 3 Faculty of Health Sciences, University of Potsdam, 14476 Potsdam, Germany

**Keywords:** Neuroplasticity, Near-infrared spectroscopy, Whole-brain, Table tennis, Neural efficiency, Unconstrained environments, Threshold-free cluster enhancement

## Abstract

There is a growing interest to understand the neural underpinnings of high-level sports performance including expertise-related differences in sport-specific skills. Here, we aimed to investigate whether expertise level and task complexity modulate the cortical hemodynamics of table tennis players. 35 right-handed table tennis players (17 experts/18 novices) were recruited and performed two table tennis strokes (forehand and backhand) and a randomized combination of them. Cortical hemodynamics, as a proxy for cortical activity, were recorded using functional near-infrared spectroscopy, and the behavioral performance (i.e., target accuracy) was assessed via video recordings. Expertise- and task-related differences in cortical hemodynamics were analyzed using nonparametric threshold-free cluster enhancement. In all conditions, table tennis experts showed a higher target accuracy than novices. Furthermore, we observed expertise-related differences in widespread clusters compromising brain areas being associated with sensorimotor and multisensory integration. Novices exhibited, in general, higher activation in those areas as compared to experts. We also identified task-related differences in cortical activity including frontal, sensorimotor, and multisensory brain areas. The present findings provide empirical support for the neural efficiency hypothesis since table tennis experts as compared to novices utilized a lower amount of cortical resources to achieve superior behavioral performance. Furthermore, our findings suggest that the task complexity of different table tennis strokes is mirrored in distinct cortical activation patterns. Whether the latter findings can be useful to monitor or tailor sport-specific training interventions necessitates further investigations.

**Supplementary Information:**

The online version contains supplementary material available at 10.1007/s10548-023-00963-y.

## Introduction

In recent years, there is mounting evidence that high-level sports performance does not only require well-developed physical capabilities but also relies on superior cognitive performance levels (Scharfen & Memmert 2019; Yarrow et al. 2009). The latter is a crucial element for extraordinary motor control being observed in (elite) athletes (Yarrow et al. 2009). To better understand the superior motor control of (elite) athletes, neural processes controlling the execution of sports-specific motor tasks were recently investigated (Nakata et al. 2010; Seidel-Marzi & Ragert 2020; Yarrow et al. 2009). To study the neural processes of athletes in a realistic and natural environment (e.g., during the execution of sport-specific motor tasks such as a table tennis stroke), mobile neuroimaging techniques such as electroencephalography (EEG) and functional near-infrared spectroscopy (fNIRS) have been applied (Carius et al. 2021; Park et al. 2015; Stephane Perrey & Besson 2018). While both EEG and fNIRS have yielded valuable insights into the neural processes being associated with motor control of sport-specific motor tasks, EEG suffers from the vulnerability to motion artifacts and a limited spatial resolution, although it provided an excellent temporal resolution as compared to fNIRS (Stephane Perrey & Besson, 2018; Seidel-Marzi & Ragert 2020). In contrast, fNIRS offers a relatively high spatial resolution, an acceptable temporal resolution, and is relatively robust against motion artifacts (as compared to EEG). Hence, it is well-situated to study motor control-related neural processes during the execution of sport-specific gross-motor tasks (Seidel-Marzi & Ragert 2020). FNIRS is a non-invasive neuroimaging technique that is based on the theory of neurovascular coupling and optical spectroscopy (for review please see Herold et al. 2018; Leff et al. 2011). In brief, fNIRS allows for an indirect assessment of cortical activity patterns by using activity-related changes in oxygenated (HbO) and deoxygenated hemoglobin (HbR) as proxies of brain activity changes (Herold et al. 2018; Leff et al. 2011). More specifically, higher cortical activity in a distinct brain area is typically reflected by an increase in HbO and a concomitant decrease in HbR resulting from an activity-related rise in the regional blood flow (Herold et al. 2018; Leff et al. 2011).

Within the sports context, our and other research groups have successfully applied fNIRS during sport-specific motor tasks such as balancing (Herold et al. 2017a, b; Seidel et al. 2017; Seidel-Marzi et al. 2021), barbell squats (Kenville et al. 2017), juggling (Carius et al. 2016), climbing (Carius et al. 2020a), basketball (Carius et al. 2020b), and table tennis (Balardin et al. 2017; Carius et al. 2021). In this context, some of the above-mentioned studies reported expertise-related differences in cortical activity patterns (Carius et al. 2016, 2021) which is, at least partly, in line with the *neural efficiency hypothesis* postulating that experts show a more efficient cortical processing (lower neural resources) than non-experts (Neubauer & Fink 2009). Recently, we investigated expertise-related differences in the behavioral performance of forehand and alternating forehand and backhand strokes as well as the corresponding cortical activation patterns in table tennis experts and novices (Carius et al. 2021). In this study, however, contrary to the neural efficiency hypothesis, we observed a more pronounced increase in cortical activation (operationalized by changes in HbO) in motor control-related brain areas such as primary motor cortex (M1), premotor cortex (PMC), and inferior parietal cortex (IPC) in experts as compared to novices regardless of the stroke technique (Carius et al. 2021). Moreover, although this study provided valuable insights into the differences in motor control-related neural processes between table tennis experts and novices in a relatively naturalistic environment, some points somewhat weaken, from a scientific perspective, the robustness and generalizability of our findings (Carius et al. 2021). In particular, in the above-mentioned study (i) the movement frequency was not standardized across conditions as inherent in a naturalistic setting (ii) fNIRS was only recorded using a regions-of-interest (ROI) based approach covering exclusively motor-related brain regions (Carius et al. 2021). Based on the evidence that a faster movement frequency in specific motor tasks (i.e., finger tapping) lead to higher activation of motor-related brain areas (Guérin et al. 2021; Kuboyama et al. 2004, 2005), the observation of a higher cortical activation in motor-related brain areas in table tennis experts in our previous study might be related to the fact that table tennis experts exhibited a higher movement frequency (i.e., a higher number of executed table tennis strokes in a specific time interval) as compared to table tennis novices (Carius et al. 2021). Furthermore, given (i) that in gross-motor tasks (i.e., walking) a slower movement frequency leads to a higher activation of the prefrontal areas (Guérin et al. 2022), and (ii) that our fNIRS setup in the previous study (Carius et al. 2021) only covered motor-related areas which, in turn, does not allow to assess of “compensational” brain activity patterns (e.g., in the prefrontal cortex), the findings of our previous study needs to be confirmed and substantiated by addressing the above-mentioned methodological limitations. Such confirmatory research (e.g., replication studies) is important to move the field of sports science forward (Halperin et al. 2018).

This assumption is further supported by the facts that (a) in table tennis athletes available neuroimaging studies investigating expertise-related effects (i.e., neural efficiency) are mostly limited to laboratory-based investigations in which no table tennis-specific movements were performed (Guo et al. 2017; Hülsdünker et al. 2019b, 2019a; Yingying Wang et al. 2019; Wolf et al. 2014; Wolf et al. 2015), and (b) that a profound knowledge on the neural process of motor control (e.g., expertise-related neural signatures of different table tennis stroke techniques) is an important prerequisite to optimize sport-specific training (Stephane Perrey & Besson 2018; Seidel-Marzi & Ragert 2020; Yarrow et al. 2009), further studies elucidating the neural processes of high-level motor performance are needed to gain a more nuanced understanding of motor control in general and to facilitate sportive success in particular. Thus, in the current study, we aimed to investigate expertise-related effects concerning different table tennis stroke techniques while recording cortical activity via a whole-head fNIRS setup in table tennis experts and novices. Based on our previous study (Carius et al. 2021), we hypothesize (i) that table tennis experts outperform novices on behavioral performance levels and (ii) that table tennis experts show altered cortical activation patterns as compared to novices. In accordance with the neural efficiency hypothesis predicting a task-related economization of brain activation patterns in experts (Ludyga et al. 2016; Neubauer & Fink 2009), we assume that table tennis novices in comparison to table tennis experts have a lower degree of neural efficiency being reflected by a higher activation of compensational (i.e., prefrontal cortex) and task-relevant brain areas (i.e., motor cortex) during table tennis strokes. Although our assumption regarding the motor-related brain activation patterns is in contrast to the findings of our study, our previous observation of a higher activation of motor-related brain areas in table tennis experts during table tennis strokes (Carius et al. 2021) is probably related to the higher number of table tennis strokes conducted by the experts as movement frequency is known to influence brain activation patterns (Guérin et al. 2021). To address the limitations of our previous study (i.e., no control for movement frequency and assessment of only motor-related brain regions), we rigorously controlled the movement frequency by using a robotic device and extended our measurement setup from motor-related areas to a whole-brain configuration. In addition, as recommended in recent best-practice recommendations (Yücel et al. 2021), we also applied state-of-the-art analyses tools to account for potential confounders of the fNIRS signal (i.e., short-separation channels to record extracerebral hemodynamic changes).

Taken together, the current study utilizing a sophisticated and rigorous methodological approach aimed to investigate expertise-related effects of different table tennis stroke techniques on cortical activation patterns and thus will broaden our knowledge on the phenomenon of neural efficiency in sport-specific tasks (i.e., table tennis strokes).

## Material and Methods

### Participants

A total of 35 right-handed healthy volunteers (average age: 25.40 ± 0.53 years; range 21–35 years; 18 women) were included in this study. The study procedure was approved by the local ethics committee of the University of Leipzig (309/17-ek) and was conducted in accordance with the latest version of the Declaration of Helsinki. None of the volunteers reported any previous neurological, psychiatric, cardiovascular, or musculoskeletal disease or took centrally acting drugs during the time of the experiment. To ensure that both groups did not significantly differ in terms of potential confounders, (i) hours of sports per week, and (ii) hours of fine motor training per week were assessed. According to the Edinburgh Handedness Questionnaire (Oldfield 1971), all volunteers were right-handed (mean handedness score of 70.59 ± 3.30; cut-off score ≥ 50 indicated right-handedness; < 50 to >  − 50 indicate ambidextrous handedness; ≤  − 50 indicated left-handedness (Dragovic 2004)). A standardized questionnaire was used to assess a) hours of sports per week and b) hours of fine motor training per week (e.g., playing a musical instrument, knitting, handicrafts, playing video games). Out of the 35 participants included, 17 were expert table tennis players (experience: 16.17 ± 1.08 years, quarterly table tennis ranking (QTTR): 1826 ± 55.67, classified as highly trained athletes: Tier 3 McKay et al. 2022), and 18 novice table tennis players (see Table 1 for details on group demographics). The QTTR is a quarterly adjusted scoring system for ranking seasonal table tennis performance. Here, the number of points depends on wins, losses, and the number of games played. The score ranges from 800 (beginner)–2700 (highest level of expertise, i.e., national league level). A QTTR of ~ 1600 thus indicates expertise related to the regional league level. To control for possible psychological confounders, all participants assessed their attention [1 (very distracted)–10 (very attentive)], fatigue [1 (sleeping)–10 (very energetic)], and discomfort [1 (no discomfort)–10 (strong discomfort)] on a visual analog scale (VAS) both before and after the entire experiment.

**Table 1:** Group demographics

Group	Age (years)	Gender (female/male)	LQ (score)	Sports/week (hours)	Fine-motor training/week (hours)
Experts *n* = 17	25.71 ± 0.97	7/10	71.49 ± 3.67	6.68 ± 0.85	1.91 ± 0.77
Novices *n* = 18	25.11 ± 0.48	9/9	69.74 ± 5.51	6.03 ± 0.96	2.06 ± 0.78

LQ, Laterality Quotient as assessed with the Edinburgh Handedness Scale [range: − 100 (full left-handed) to + 100 (full right-handed)]. Hours of sports per week and hours of fine motor training per week (e.g., playing a musical instrument, knitting, doing handcrafts, playing video games with a keypad or joystick) were assessed with a questionnaire. All values are depicted as mean standard error (SE) of the mean. Statistical analysis revealed no differences in age, gender, LQ, sports/week, or fine-motor training/week between groups

### Experimental Procedure

**Fig. 1:**
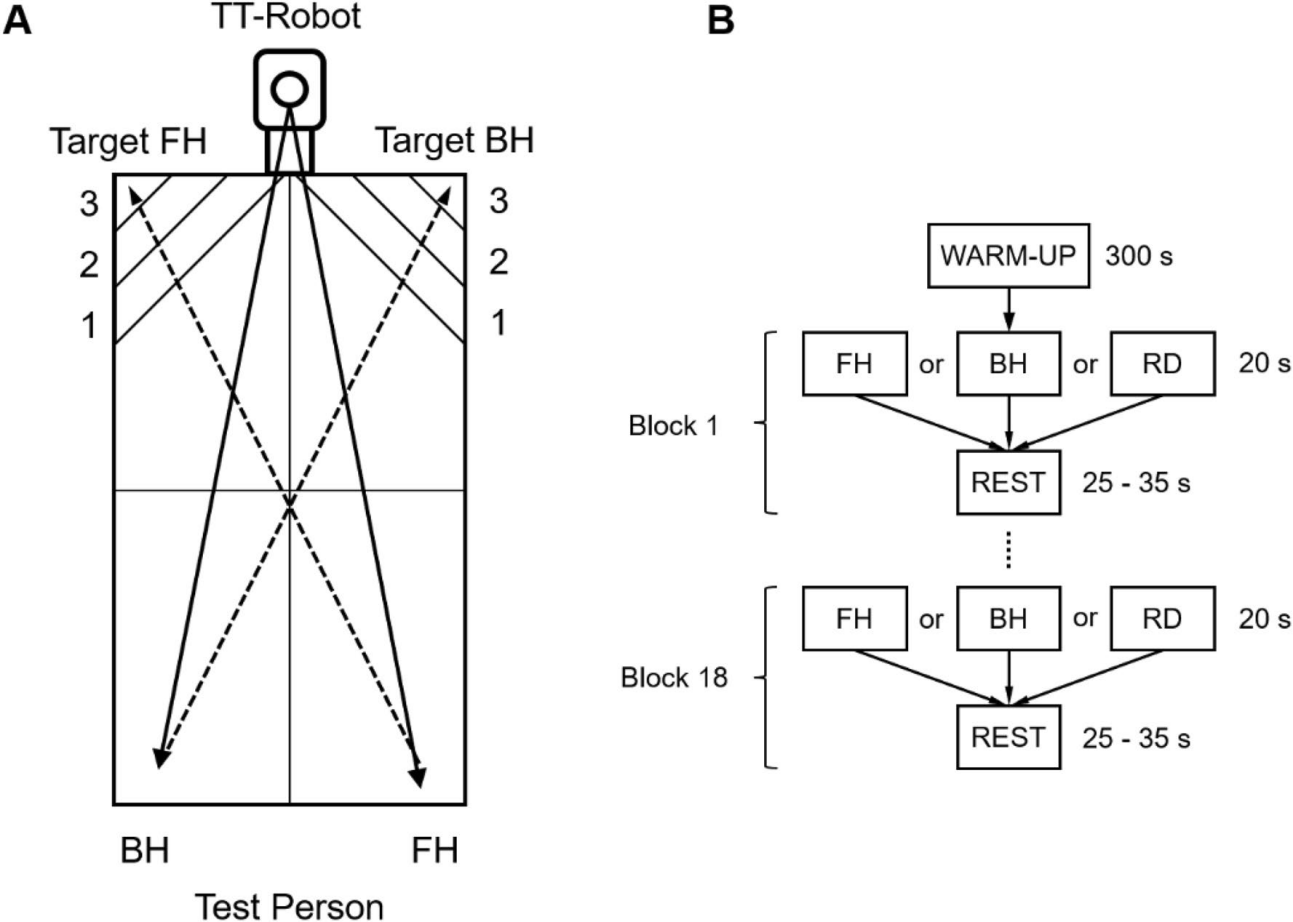
Study design and experimental setup. **A** Forehand (FH) and backhand (BH) strokes illustrated on a table tennis plate with target areas 1–3. **B** Experimental procedure: Participants started with a 5 min warm-up phase. Following the warm-up phase, participants performed FH and BH strokes, as well as the randomized (RD) execution of forehand and backhand strokes with the dominant right hand

The present study aimed to compare cortical activity patterns between table tennis experts and novices during the execution of forehand (FH) and backhand (BH) strokes, as well as the randomized (RD) execution of forehand and backhand table tennis strokes. For this purpose, the participants performed FH cross-court, BH cross-court, and RD cross-court strokes against topspin balls played by an app-controlled table tennis robot (Donic Newgy Robo-Pong 3050XL, Germany) in a standardized manner. Standardization includes the spin type (topspin), spin strength/ball speed, placement of the balls on the table, height, number of balls (13) and wait time (1.54 s). FH, BH, and RD strokes were each executed according to a block design for 6 × 20 s in random order (jittered intertrial interval 25–35 s, see Fig. 1b), whereby the participants were instructed to perform strokes as accurately as possible. The target areas on the FH and BH sides were isosceles right triangles with side lengths of 25 cm (3 points), 50 cm (2 points), and 75 cm (1 point), respectively (see Fig. 1). Target accuracy was recorded with a high-speed video camera and evaluated offline. To control for movement speed, we used a 9-axis accelerometer combining a 3-axis gyroscope, 3-axis accelerometer and 3-axis magnetometer, which is an integral part of the fNIRS system (NIRSport2, NIRX, US, 100 Hz). The accelerometer was fixed to the upper arm using an elastic strap (Velcro strap, Noraxon, Scottsdale, US) and a cap holder designed for NIRS samples and accelerometers.

The onsets of the FH, BH, and RD blocks were presented as auditory stimuli via Psychopy (Peirce 2007). In addition, fNIRS triggers were set via Psychopy.

### Functional Near-Infrared Spectroscopy (fNIRS)

**Fig. 2:**
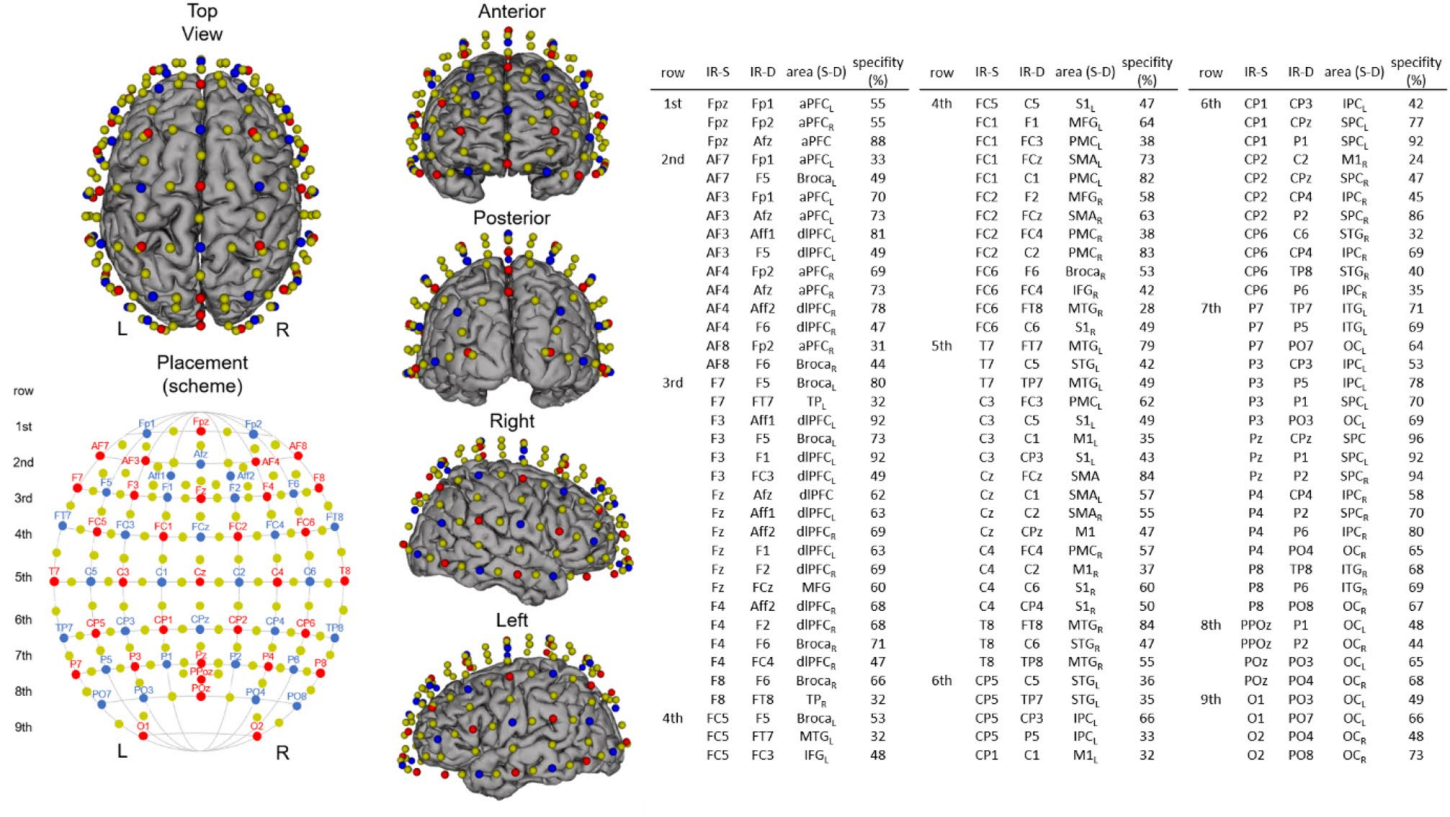
Illustration of fNIRS configuration used during table tennis. Sources are shown as red dots and detectors as blue dots. Yellow dots represent each center of the 108 channels (inter-optode distance 3 cm). 10–20 EEG positions for infrared sources (IR-S) and detectors (IR-D), respective brain regions (arranged in rows), targeted by a 10–20 system transfer method and defined by the “Brodmann” Atlas (*aPFC* anterior prefrontal cortex, *Broca* broca area, *dlPFC* dorsolateral prefrontal cortex, *IFG* inferior frontal gyrus, *IPC* inferior parietal cortex, *ITG* inferior temporal gyrus, *M1* primary motor cortex, *MFG* middle frontal gyrus, *MTG* middle temporal gyrus, *OC* occipital cortex, *PMC* premotor cortex, *S1* primary somatosensory cortex, *SMA* supplementary motor cortex, *SPC* superior parietal cortex, *STG* superior temporal gyrus, *TP* temporal pole; *L* left hemisphere, *R* right hemisphere, Zimeo Morais et al. 2018)

Hemodynamic responses were recorded on both hemispheres using a whole-brain continuous-wave fNIRS system (NIRSport2, NIRX, US). The fNIRS setup used 32 LED light sources and 32 avalanche photodiode detectors with an inter-optode distance of approximately 30 to 40 mm (depending on specific channel configuration and head circumference of the individual participant), which form 108 actual measurement channels. For an illustration of the fNIRS setup see Fig. 2. Fixation of a source and detector distance was achieved by using so-called distance holders. The NIRSport2 measures simultaneously at wavelengths of 760 nm and 850 nm and uses time and frequency multiplexing to minimize crosstalk between wavelengths and optodes. FNIRS optode placement was performed using an fNIRS cap (with different sizes) which ensures standardized sensor placement according to the well-established 10–20 EEG system.

In addition to the 108 (standard) channels, we used a short-distance detector bundle (NIRx Medical Technologies, Glen Head, NY) to eliminate potential fNIRS confounders, such as extra-cerebral blood flow alterations. For that purpose, we used additional short-distance detectors with an inter-optode distance of 8 mm, as opposed to the inter-optode distance for all other long-separation channels of our configuration (i.e., between ca. 30 and 40 mm). This resulted in eight short-distance channels, which were taken into account in the analysis of the fNIRS signal (see Analysis). Data were acquired with a sampling frequency of 10.1725 Hz. To rule out expertise-related differences, we measured cardiac stress, operationalized via heart rate, during the execution of table tennis strokes using an Apple Watch (Series 6) with a sampling frequency of 1 Hz.

### Data Analyses

#### Hemodynamics

FNIRS data analysis was performed in MATLAB (MathWorks, Natick, MA, United States of America) using HOMER3 (version 1.35.11) (Huppert et al. 2009) and QT-NIRS (Hernandez & Pollonini 2020). Statistical analysis was performed using the TFCE-Toolbox (Mensen & Khatami 2013).The first step in the fNIRS signal pre-processing was channel pruning. In this work, we used QT-NIRS (Hernandez & Pollonini 2020), a MATLAB-based tool for estimating the quality of an fNIRS signal based on physiology-related measures that are independent of the specific instrument and of the experimental paradigm being used. This approach, first proposed by Pollonini et al. (2016), uses a combination of time-domain (scalp coupling index, SCI) and frequency-domain measures (peak spectral power, PSP) of the strength of the systemic pulsation to evaluate both optode-scalp coupling and the presence of movement artifacts at all time points in a dataset [F_minmax_ = (0.5 2.5); wLength = 3; sciThld = 0.7; pspThld = 0.1]. If more than 30% of channels from a given dataset were deemed invalid, then the whole dataset was excluded from further analysis. Following this procedure, 13 subjects had to be excluded. The remaining participants (*n* = 22, 11 experts and 11 novices) show good data quality for 82.1% of the channels. Second, the raw intensity signals were converted to changes in optical density (Huppert et al. 2009).

Correction for motion artifacts was performed using wavelet filtering (Brigadoi et al. 2014; Di Lorenzo et al. 2019). We used the algorithm described by Molavi and Dumont (2012) as implemented in the HOMER3 hmrR_MotionCorrectWavelet filtering function (inter-quartile range 1.219, Carius et al. 2020a). Following motion-artifact correction, the data was slightly low-pass filtered using 3 Hz as low pass cutoff frequency. We did not use a high pass filter as the following GLM handles this with a polynomial drift correction.

Attenuation changes of both wavelengths (850 nm and 760 nm) were transformed to concentration changes of oxy- and deoxygenated hemoglobin (HbO and HbR, respectively) using the modified Beer-Lambert approach (partial pathlength factor: 6.0; Huppert et al. 2009). As recommended in the current literature (Yücel et al. 2021), we report HbO and HbR (instead of only reporting changes in one chromophore), however, as concentration changes of HbR are considered to (i) be influenced by a lower extent by systemic physiological noise (Dravida et al. 2018; Kirilina et al. 2012), (ii) have a stronger correlation with the blood oxygen level-dependent signal of the functional magnetic resonance imaging (Huppert et al. 2006a; Huppert et al. 2006b), and (iii) are spatially more focused (Dravida et al. 2018; Plichta et al. 2007), we primarily, but not solely, focus on this parameter in the presentation and interpretation of the results.

In order to regress extra-cerebral contaminations (measured by short-distance channels) out of the signal, we modeled the hemodynamic response function (HRF) by using a general linear model approach (GLM) that uses ordinary least squares and a consecutive sequence of Gaussian functions with a standard deviation of 0.5 s and their means separated by 0.5 s over a specific regression time (used parameters in HOMER3 hrmR_GLM function: trange − 2.0 to 40; glmSolveMethod 1; idxBasis 1; paramBasis 0.5 and 0.5). Furthermore, to account for baseline drift, we used a third order polynomial fit. As implemented in this function, short-separation regression (SSR) is performed with the short-separation channel, which shows the highest correlation with the respective long-separation channel (Lühmann et al. 2020; Yücel et al. 2015). Single trials were baseline corrected (regarding 2 s before stimulus onset) and time courses of HbO and HbR concentration changes in each measurement channel and condition (FH, BH, RD) were block-averaged. The entire time courses of HbO and HbR were exported for TFCE analysis.

#### Behavioral Data

The target accuracy of FH, BH, and RD was recorded with a high-speed video camera (iPhone 13, 4096 × 2160 Pixel (4 K), 60 frames per second, Apple Inc., California) and further evaluated offline by two experimenters. For this purpose, the hit points of the table tennis balls on the board had to be assigned to the respective target areas (isosceles right triangles with side lengths of 25 cm (3 points), 50 cm (2 points), and 75 cm (1 point), see Fig. 1). Total points were determined for each stroke condition.

The analysis of the movement speed was performed with a customized Matlab script. The three components of the gyroscope were used for this purpose. The assignment of gyroscope data to the table tennis stroke movements was based on recorded fNIRS triggers. Using the local maxima of the three components of the gyroscope, the maximum angular velocities of the upper arm were determined. The angular velocities of the 18 strokes were averaged block wise for each subject and each stroke condition. The component with the highest angular velocities represents the main movement in the sagittal plane of motion and was used to evaluate expertise- and task-related differences.

### Statistical Analyses

In fNIRS studies, the assumptions for parametric tests are often violated (e.g., normal distribution). Furthermore, due to mass univariate testing, the multiple comparison problem (MCP) arises, especially when using whole-brain configurations with a huge number of channels. For these reasons, nonparametric threshold-free cluster enhancement (TFCE) was applied with a cluster threshold of p = 0.05 (Mensen & Khatami 2013; Smith & Nichols 2009) and 10.0000 permutations. Data-driven cluster-based permutation tests are widely used in ERP/ERF analysis (EEG/MEG) and have also been applied in fNIRS studies as a suitable solution strategy for the problems mentioned above (Abboub et al. 2016; Ferry et al. 2016; Mahmoudzadeh et al. 2013). Cluster-based methods are particularly useful for the statistical analysis of data where spatial and/or temporal dependencies are expected, as in the case of EEG or fNIRS data. TFCE tests were conducted in Matlab using the TFCE-Toolbox (Mensen & Khatami 2013). Differences in HbO and HbR concentration changes between FH, BH, and RD (task as within-subject-factor), respectively, novices and experts (group as between-subject-factor) were tested using TFCE mixed two-way ANOVA (default TFCE parameters: E = 2/3, H = 1, Mensen & Khatami 2013; Smith & Nichols 2009). In the case of non-significant interactions, we conducted post hoc tests for the main effects task and expertise. For the factor task, we used one-way repeated measures TFCE ANOVA. For factor expertise we used independent sample TFCE T-Tests (default parameters: E = 2/3, H = 2). The TFCE statistic does not provide an estimate of the effect size. Estimating the effect size using the T-statistics only takes into account the magnitude of the effect, but does not take into account the contribution of the cluster size to the TFCE statistic.

The resulting spatio-temporal clusters are printed as color-coded T- & F-Maps in time (− 2 to 40 s) and space (108 channels). We report the actual mean differences, cluster sizes (the number of significant channels and/or time points), corrected p-values, and TFCE values for peak coordinates of the resulting clusters. In addition, to illustrate the task-related changes during the execution of the table tennis strokes, we averaged the TFCE test values from 5 to 20 s and mapped them onto the brain surface using the Brain Function Mapping Tool of Wang et al. (2016). Finally, TFCE test values were summed in time and space according to their associated brain areas, separately for left and right hemispheres. In the results section, L represents left and R represents right hemisphere.

The behavioral data (target accuracy & movement) of the FH, BH, and RD were analyzed using a mixed ANOVA with post-hoc tests (i.e., T-tests). The threshold for statistical significance was set to α = 0.05.

## Results

### Behavioral Data

**Fig. 3:**
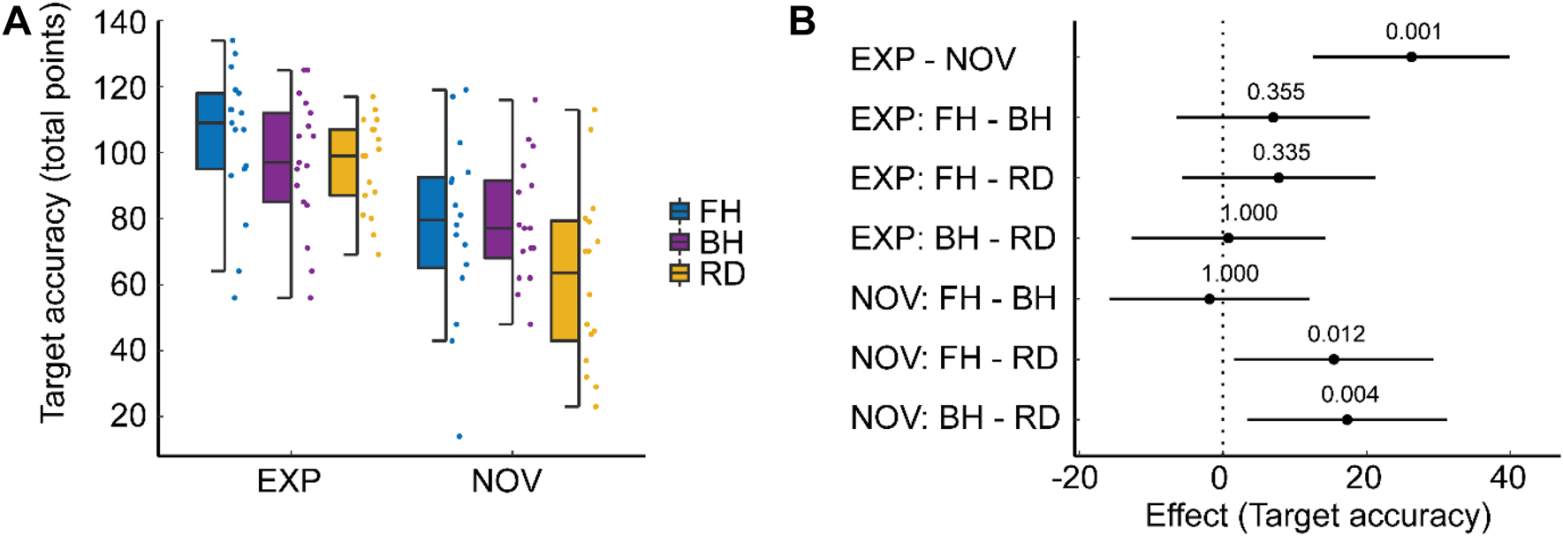
Group and task effects on target accuracy during table tennis. Harrell plot combining box plot, dot plot and forest plot. A Box plot and dot plot showing target accuracy of Novices and Experts executing Forehand (FH), Backhand (BH) and Forehand/Backhand strokes (RD). B Forest plot showing modeled effects. Experts achieved higher target accuracy as compared to novices (EXP-NOV). Within experts, target accuracy did not differ between conditions (EXP: FH-BH, FH-RD, BH-RD, Holm adjusted p-values). In contrast, novices achieved lower target accuracy in RD as compared to FH and BH (NOV: FH-BH, FH-RD, BH-RD, Holm adjusted p-values)

In general, experts achieved higher target accuracy as compared to novices [mean ± SD: NOV: 74.20 ± 25.18 points, EXP: 99.48 ± 19.48; *F*(1, 31) = 15.36, *p* < 0.001, *η*_*p*_^*2*^ = 0.33, Fig. 3].

Furthermore, we also identified task differences across groups (FH: 91.18 ± 27.74, BH: 88.49 ± 21.28, RD: 79.70 ± 27.30; *F*(1.52, 47.16) = 7.39, *p* = 0.004, *η*_*p*_^*2*^ = 0.19). There was no interaction between task and group [*F*(1.52, 47.16) = 3.41, *p* = 0.054, *η*_*p*_^*2*^ = 0.10]. Within experts, target accuracy did not differ between conditions (FH vs. BH: *t*(16) = 1.59, *p*_*holm*_ = 0.354, *d* = 0.32; FH vs. RD: *t*(16) = 1.76, *p*_*holm*_ = 0.335, *d* = 0.35; BH vs. RD: *t*(16) = 0.17, *p*_*holm*_ = 1.000, *d* = 0.04). In contrast, novices achieved lower target accuracy in RD as compared to FH and BH (FH vs. BH: *t*(15) = -0.41, *p*_*holm*_ = 1.000, *d* = − 0.09; FH vs. RD: *t*(15) = 3.39, *p*_*holm*_ = 0.012, *d* = 0.70; BH vs. RD: *t*(15) = 3.80, *p*_*holm*_ = 0.004, *d* = 0.79). The aforementioned results for target accuracy are based on the total sample (*n* = 35). The statistical analysis of the fNIRS subsample (*n* = 22) leads to the same behavioral results as the analysis of the total sample (see Supplementary Table 1).

In addition, we quantified expertise-related differences in movement speed. There were no differences in movement speed between experts and novices (mean ± SD: NOV: 26.33 ± 13.23°s^−1^, EXP: 35.92 ± 8.10°s^−1^; *F*(1, 18) = 3.83, *p* = 0.066, *η*_*p*_^*2*^ = 0.18) and no interaction between task and group (*F*(2, 36) = 1.31, *p* = 0.282, *η*_*p*_^*2*^ = 0.07). In contrast, we identified task differences across groups (FH: 33.48 ± 15.86, BH: 23.44 ± 12.09, RD: 36.48 ± 14.77; *F*(2, 36) = 9.40, *p* < 0.001, *η*_*p*_^*2*^ = 0.34). Here, BH was performed at a lower movement speed compared to FH and RD (FH vs. BH: *t*(19) = 3.19, *p*_*holm*_ = 0.006, *d* = 0.74; RD vs. BH: *t*(19) = 4.14, *p*_*holm*_ < 0.001, *d* = 0.96; FH vs. RD: *t*(19) = − 0.95, *p*_*holm*_ = 0.347, *d* = − 0.22).

### Hemodynamics

Nonparametric cluster-based permutation analysis (TFC E) revealed no interactions between groups (experts vs. novices) and conditions (FH vs. BH vs. RD), neither for HbO (peak significance found at PMC_L_: *F*(2,40)_*max*_ = 9.41, *p*_*max*_ = 0.434, FWE TFCE-corrected), nor for HbR (peak significance An39_R_: *F*(2,40)_*max*_ = 9.17, *p*_*max*_ = 0.582, FWE TFCE-corrected). In contrast, testing main effects, cluster-based permutation tests indicated differences between groups and conditions suggesting that expertise level (novices vs. expert) and task complexity (i.e., FH vs. BH vs. RD) modulate the cortical hemodynamics. In the following, we will describe our results of expertise- and task-related effects on cortical hemodynamics in more detail.

#### Effect of Expertise

**Fig. 4:**
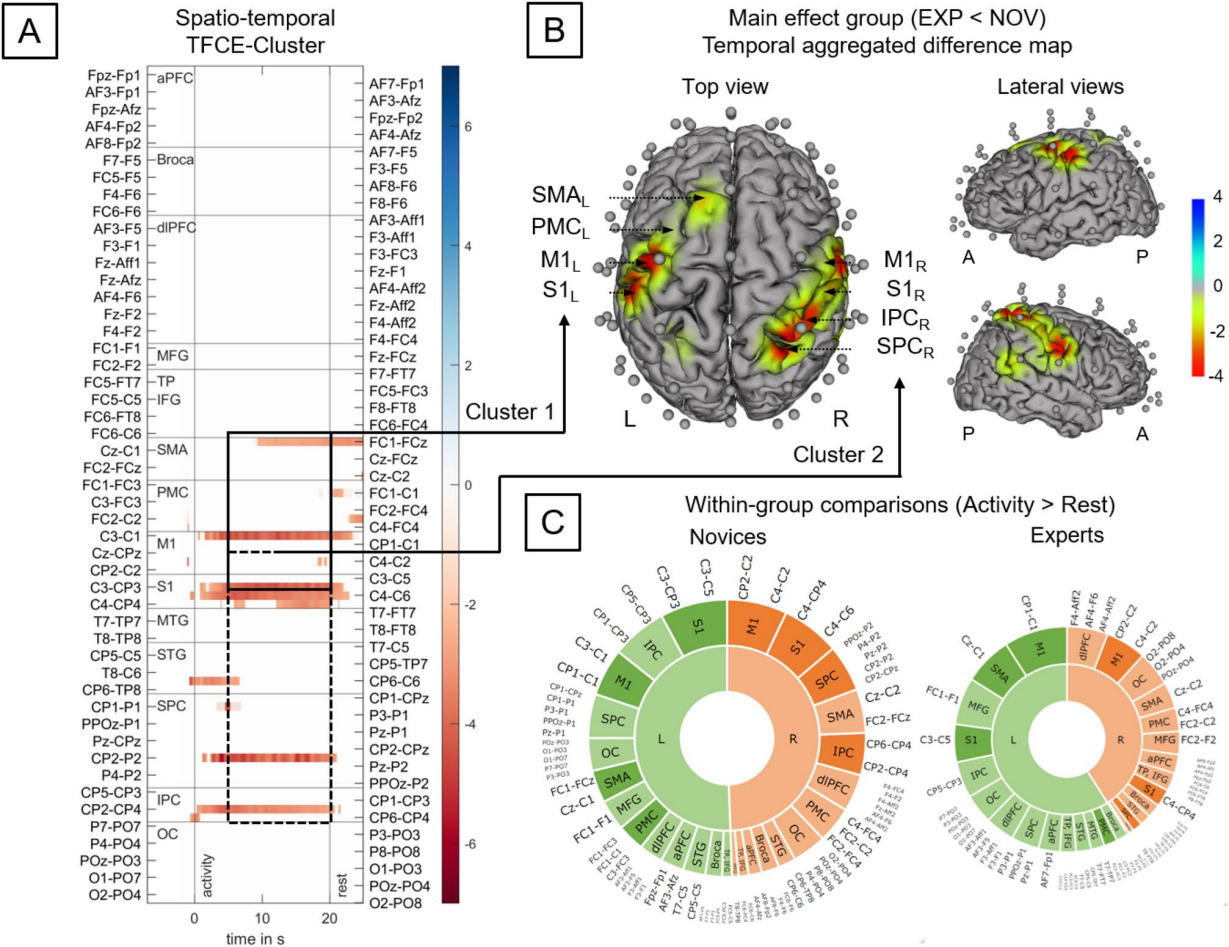
Group effects on deoxygenated hemoglobin (HbR) concentration changes during table tennis [Novice (NOV) vs. Experts (EXP) executing forehand and backhand strokes] according to TFCE analysis. A Raster diagram showing significant data points (spatio-temporal cluster). Rectangles indicate channel/time points modulated by expertise. Red rectangles indicate higher concentration changes for novices. The colorbar indicates TFCE t-values. Note that channels (source-detector combinations) are organized along the y-axis according to their associated brain areas. B Temporal aggregated difference map (EXP—NOV). Optodes (sources and detectors) are shown for the topographic images; colors represent mean TFCE t-values (sample range 5–20 s). Images are thresholded at p < 0.05 (Top view, Cluster 1: 22 channels including SMA_L_, PMC_L_, M1_L_, S1_L_, time range 0–40 s, *t*(20)_max_ = − 5.02, *p*_max =_ 0.001, Cluster 2: 9 channels including M1_R_, S1_R_, IPC_R_, SPC_R_, time range 0–23 s, *t*(20)_max_ =  − 5.83, *p*_max =_ 0.005). All channels indicate higher concentration changes in HbR for novices. C Within-group comparisons activity vs. baseline (rest) concentration changes. Sunburst plots showing novices resp. experts. Values are summed TFCE t-values (sample range 5–20 s) for all significant channels resp. associated brain areas. The size of the segments reflects the sum of the TFCE t-values of the respective brain areas. The total size of the sunburst plots reflects the total sum of all significant TFCE t-values. Please note, that all channels show higher concentration changes during activity compared to baseline. *TFCE* threshold-free cluster enhancement, *A* Anterior, *P* Posterior, *L* left hemisphere, *R* right hemisphere, *aPF* anterior prefrontal cortex, *Broca* broca area, *dlPFC* dorsolateral prefrontal cortex, *IFG* inferior frontal gyrus, *IPC* inferior parietal cortex, *ITG* inferior temporal gyrus, *M1* primary motor cortex, *MFG* middle frontal gyrus, *MTG* middle temporal gyrus, *OC* occipital cortex, *PMC* premotor cortex, *S1* primary somatosensory cortex, *SMA* supplementary motor cortex, *SPC* superior parietal cortex, *STG* superior temporal gyrus, *TP* temporal pole

Regarding HbR, the difference between experts and novices (Fig. 4a) was driven by two clusters primarily located over left (Cluster 1) and right pre- and postcentral channels (Cluster 2). Cluster 1 included left SMA, PMC, M1, S1 (22 unique channels; time range 0–40 s; mean ± SD: NOV: 19.56 ± 14.03 µM mm, EXP: 13.70 ± 16.94 µM mm; *t*(20)_max_ =  − 5.02, *p*_max_ = 0.001, FWE TFCE-corrected, Fig. 4b). Cluster 2 included right M1, S1, IPC, SPC and STG (9 unique channels; time range 0–23 s; NOV: 30.69 ± 12.59 µM mm, EXP: 1.62 ± 9.64 µM mm; *t*(20)_max_ =  − 5.83, *p*_max_ = 0.005, FWE TFCE-corrected). All the aforementioned channels showed higher concentration changes in HbR in novices compared to experts. TFCE-based post hoc testing for novices revealed a significant decrease in HbR concentration during table tennis compared to baseline in bilateral M1, S1, SPC, SMA_L_, PMC_L_ and IPC_R_ (*t*(32)_max_ = − 9.42, all channels: *p*_max_ < 0.002, Fig. 4c shows summed TFCE t-values). In contrast, in experts a more focused task-related decrease in HbR in bilateral M1, PMC_L_ and S1_R_ was identified [*t*(32)_max_ = − 7.35, *p*_max_ < 0.001].

Considering HbO, TFCE identified two spatio-temporal clusters comparing experts and novices (suppl. Fig. 1a). Cluster 1 includes left dlPFC and left Broca [two unique channels, time range 12–23 s; NOV: 34.53 ± 5.47 µM mm, EXP: 102.68 ± 7.54 µM mm; *t*(20)_max_ = 4.43, *p*_max_ = 0.039]. Cluster 2 includes bilateral dlPFC, MFG, SMA, PMC, M1, S1, SPC, IPC and OC (54 unique channels, time range 0–40 s; NOV: 22.15 ± 26.02 µM mm, EXP: − 50.76 ± 27.75 µM mm; *t*(20)_max_ = − 7.47, *p*_max_ < 0.001). In the time range of 5–20 s only bilateral dlPFC, MFG, SMA, PMC & M1 were included (17 channels, Supplementary Fig. 1b). For all channels belonging to cluster 2, higher concentration changes in HbO were found in novices than in experts. TFCE-based post hoc testing for novices showed significant increases in concentration changes in HbO during table tennis compared to baseline in bilateral dlPFC, MFG, SMA, PMC, M1 and Broca_L_ [*t*(32)_max_ = 9.15, all channels: *p*_max_ < 0.001, see Supplementary Fig. 1c]. Experts showed increases in task-related concentration changes in HbO in left Broca, left dlPFC and left M1 [*t*(32)_max_ = 8.63, *p*_max_ < 0.001]. In contrast, in the right hemisphere MFG, SMA, PMC and M1 showed decreases (*t*(32)_max_ = − 6.79, *p*_max_ < 0.004).

#### Effect of Task

**Fig. 5:**
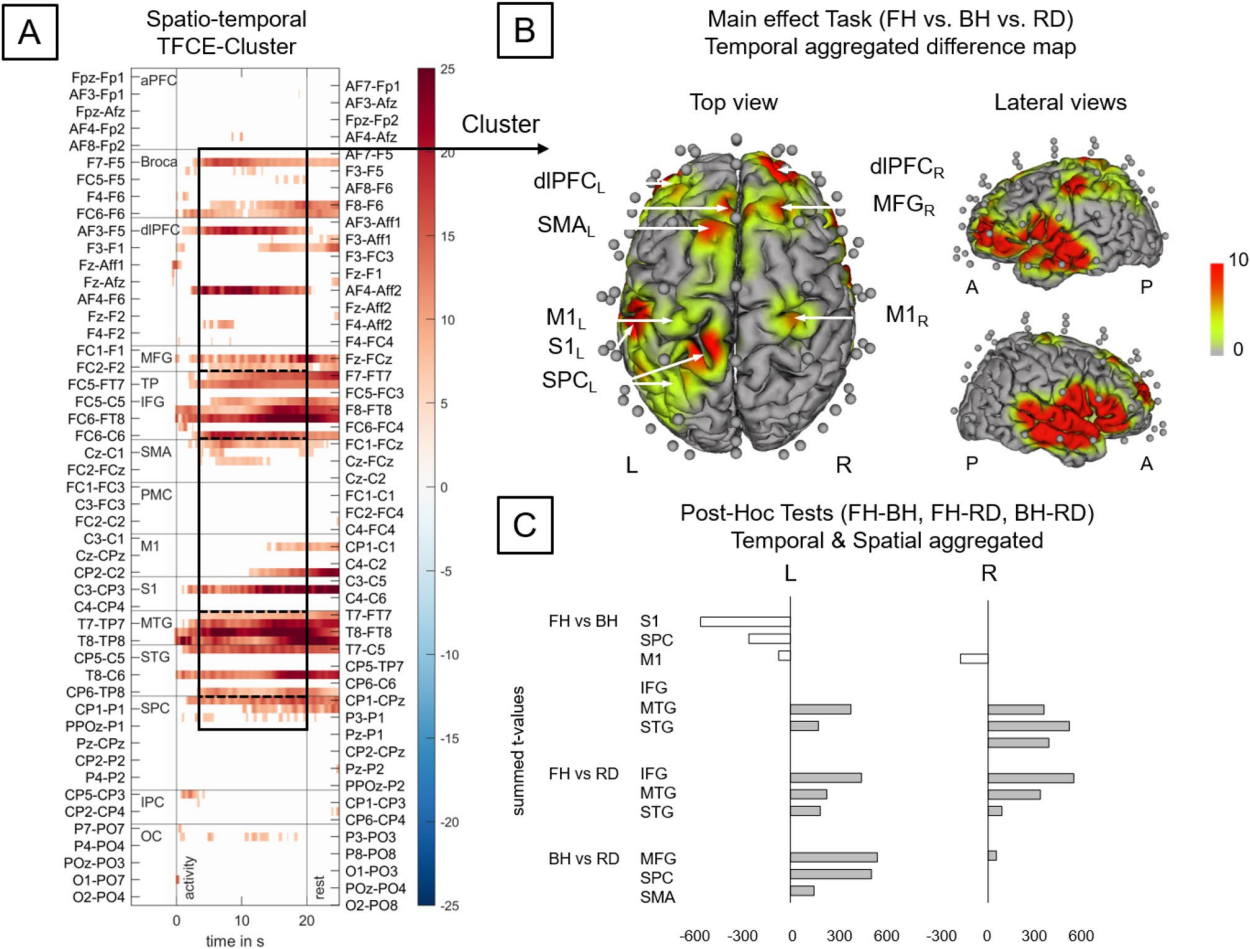
Task effects on deoxygenated hemoglobin (HbR) concentration changes during table tennis (Forehand vs. backhand vs. Forehand/backhand strokes across groups) according to TFCE analysis. A Raster diagram showing significant data points (spatio-temporal cluster). Rectangles indicate channel/time points modulated by task. The colorbar indicates TFCE f-values. Note that channels (source-detector combinations) are organized along the y-axis according to their associated brain areas (Zimeo Morais, Balardin, & Sato 2018). B Temporal aggregated difference map (FH vs. BH vs. RD). Optodes (transmitters and detectors) are shown for the topographic images; colors represent mean TFCE f-values (sample range 5–20 s). Images are thresholded at p < 0.05 (Top view, Cluster 1: 68 channels including bilateral Broca, dlPFC, MFG, SMA, PMC, M1, MTG, STG, SPC, IPC as well as aPFC_L_ and S1_L_, time range 0–40 s, *F*(2,42)_max_ = 29.76, *p*_max_ < 0.001). C Post-hoc Tests FH—BH, FH—RD and BH—RD. Bars represent post-hoc comparisons between FH, BH & RD. Values are summed TFCE t-values (sample range 5–20 s) for all significant channels belonging to the aforementioned cluster resp. brain areas. *TFCE* threshold-free cluster enhancement, *A* Anterior, *P* Posterior, *L* left hemisphere, *R* right hemisphere, *aPFC* anterior prefrontal cortex, Broca broca area, *dlPFC* dorsolateral prefrontal cortex, *IFG* inferior frontal gyrus, *IPC* inferior parietal cortex, *ITG* inferior temporal gyrus, *M1* primary motor cortex, *MFG* middle frontal gyrus, *MTG* middle temporal gyrus, *OC* occipital cortex, *PMC* premotor cortex, *S1* primary somatosensory cortex, *SMA* supplementary motor cortex, *SPC* superior parietal cortex, *STG* superior temporal gyrus, *TP* temporal pole

Regarding HbR, the difference between conditions (FH vs. BH vs. RD) was mainly driven by a widespread cluster containing pre- and postcentral channels as well as temporal subcluster on both hemispheres (see Fig. 5). This cluster included bilateral dlPFC, MFG, SMA, PMC, M1, MTG, STG, SPC, IPC and S1_L_ [68 unique channel, time range 0–40 s; *F*(2,42)_*max*_ = 29.76, *p*_*max*_ < 0.001, FWE TFCE-corrected]. During the execution of the table tennis strokes only bilateral dlPFC, PMC, M1, MTG, and STG as well as MFG_R_, SMA_L,_ S1_L_, SPC_L,_ and IPC_L_ were included (Fig. 5b). Furthermore, there was a small cluster including aPFC and dlPFC [three unique channel, time range 2–21 s; *F*(2,42)_*max*_ = 25.29, *p*_*max*_ = 0.01, FWE TFCE-corrected].

Cluster-based post hoc pairwise comparison of FH and BH showed various differences depending on the brain area. In bilateral M1, S1_L_, and SPC_L_, we observed higher concentration changes in HbR during the execution of FH [*t*(32)_max_ = − 5.03 , all channels: *p*_*max*_ < 0.022]. In contrast, in bilateral MTG and STG as well as IFGr, we observed higher concentration changes in HbR during the execution of BH [*t*(32)_max_ = 6.08, *p*_*max*_ < 0.042, Fig. 5c shows summed TFCE t-values]. The comparison of FH and RD showed higher HbR concentrations changes in bilateral MTG, STG, and IFG during RD [*t*(32)_max_ = 5.60, *p*_*max*_ < 0.005]. With regard to the statistical comparison of BH and RD, we observed higher HbR concentrations changes in RD in bilateral MFG, SMA_L,_ and SPC_L_ [*t*(32)_max_ = 5.54, *p*_*max*_ < 0.044].

The HbO the difference between conditions (FH vs. BH vs. RD) was also driven by a single, widespread cluster containing pre- and postcentral channels as well as a contiguous temporal subcluster on both hemispheres (suppl. Figure 2a). This cluster included bilateral dlPFC, SMA, MTG, STG and SPC as well as MFG_L_, PMC_L_, M1_L_, S1_L_ and IPC_L_ [66 unique channel, time range 0–40 s; *F*(2,42)_*max*_ = 28.20, *p*_*max*_ < 0.001]. Except for IPC_L_, all of the above-mentioned areas were included during the execution of the table tennis strokes (suppl. Figure 2b). Post hoc pairwise comparison of FH and BH showed no differences. In contrast, comparing FH and RD higher HbO concentrations changes were noticed in MTG_R_, STG_R_, and IFG_R_ during RD [*t*(32) _max_ = − 4.07 , all channels: *p*_*max*_ < 0.026, suppl. Figure 2c shows summed TFCE t-values]. When comparing BH and RD, we observed higher HbO concentrations changes in RD in bilateral IFG, MTG and STG [*t*(32)_max_ = − 6.72, *p*_max_ < 0.001] as well as M1_L_ and S1_L_ [*t*(32)_max_ = − 3.73, *p*_*max*_ < 0.016].

### Psychological and Physiological Confounders

Statistical analysis of the questionnaires revealed no pre-post differences, neither for the participants’ attention level, nor for the fatigue level [attention: pre: 8.0 ± 1.0 (median ± MAD), post: 7.0 ± 1.0, z = 0.32, p = 0.755; fatigue: pre: 7.0 ± 1.0, post: 7.0 ± 1.0, z =  − 0.58, p = 0.557]. Nevertheless, regarding discomfort, there was an increase from pre- to posttest (pre: 1.0 ± 0.0, post: 2.0 ± 1.0, z =  − 3.62, p < 0.001). However, there were no statistically significant differences between experts and novices concerning pretest (attention: p = 0.111, U = 200.0, fatigue: p = 0.736, U = 142.5, discomfort: p = 0.303, U = 127.0), posttest (attention: p = 0.055, U = 96.5, fatigue: p = 0.068, U = 98.5, discomfort: p = 0.731, U = 163.5), or pre–posttest differences (fatigue: p = 0.116, U = 106.5, discomfort: p = 0.362, U = 179.5) with the exception of attention (p = 0.002, U = 62.0).

Concerning cardiac stress operationalized via heart rate recordings during the execution of the table tennis strokes, there were no difference between experts and novices (mean ± SD: NOV: 88.14 ± 10.69 bpm, EXP: 92.72 ± 11.93 bpm; *F*(1, 31) = 1.37, *p* = 0.251, *η*_*p*_^*2*^ = 0.04), no task-related differences (*F*(1.45, 45.02) = 1.51, *p* = 0.232, *η*_*p*_^*2*^ = 0.05) and no interaction between task and group (*F*(1.45, 45.02) = 1.97, *p* = 0.162, *η*_*p*_^*2*^ = 0.06). The aforementioned results (questionnaires & heart rate) are based on the total sample (*n* = 35). The statistical analysis of the fNIRS subsample (*n* = 22) leads to the same outcomes (see Supplementary Tables 2 & 3).

## Discussion

In recent years, there is an increasing interest to utilize neuroimaging methods to improve our understanding of the neural processes of sport-specific tasks (Carius et al. 2021; Seidel-Marzi & Ragert 2020). In this context, the current study aimed to investigate expertise-related effects concerning different table tennis stroke techniques while recording cortical activity via a whole-head fNIRS setup in table tennis experts and novices and to extend the findings of our previous study (Carius et al. 2021). Based on our previous study (Carius et al. 2021) and the current evidence regarding the neural efficiency hypothesis (Li & Smith 2021; Neubauer & Fink 2009), we hypothesized (i) that table tennis experts outperform novices on behavioral performance levels and (ii) that table tennis experts show a higher neural efficiency than novices which is reflected by a lower activation of task-specific (e.g., PMC, M1) and compensational brain areas (e.g., dlPFC) in experts.

In line with both of our hypotheses, we observed (i) that table tennis experts outperformed novices with respect to the target accuracy of table tennis strokes and (ii) that the superior behavioral performance of expert table tennis players was accompanied by distinct cortical activities that probably reflects a better neural efficiency. In the following, we will discuss our findings in more detail.

### Behavioral Data

Our results indicate that table tennis experts achieve a higher target accuracy than novices in FH and BH. In general, our findings are in line with previous studies showing that table tennis experts as compared to novices achieved a superior performance concerning behavioral indices of table tennis strokes (e.g., target accuracy) (Schaefer & Amico 2022; Schaefer & Scornaienchi 2019). Furthermore, the finding that only novices, in contrast to experts, exhibited a performance decrease in a more complex motor task condition (i.e., RD) implies that the increase in the complexity of the motor task is not challenging enough to pose serious demands on the motor control resources of table tennis experts to lead to measurable decrements in behavioral performance (i.e., target accuracy) or, in other words, that table tennis experts have sufficient motor control resources allowing them to cope with the increased motor task complexity. The latter might be related to a higher neural efficiency of expert athletes allowing them to solve a specific tasks with a more efficient utilization of neural resources as compared to novices (Neubauer & Fink 2009). Furthermore, our findings complement the current literature in which such expertise-related differences in motor performance (i.e., operationalized by the number of hits) were not reported for the modulation of the difficulty of an additional cognitive task in a dual-task situation (Schaefer & Scornaienchi 2019). In conjunction with our observations, the finding of Schaefer and Scornaienchi (2019) suggests that neural efficiency is relatively task-specific and do not fully generalize to a broad set of (unfamiliar) tasks (i.e., solving a cognitive task while playing table tennis), although, arguably more research is necessary, to provide further empirical support for this assumption.

The finding that BH was performed at lower movement speed compared to FH and RD might raise the assumption that BH also leads to higher target accuracy. However, this was not the case in our study. Hence, lower movements speed during TT does not seem to affect motor performance, neither in novices nor in experts.

### Hemodynamics

#### Expertise-Related Effects

To better understand expertise-related differences in sport-specific skills by probing theories aiming to explain the former phenomenon (e.g., neural efficiency hypothesis), the application of neuroimaging methods (e.g., fNIRS) is essential (Stephane Perrey & Besson 2018; Seidel-Marzi & Ragert 2020). In the current study, we observed that novices compared to table tennis experts showed more pronounced alterations in cortical activity during different table tennis strokes. In particular, we found that novices as compared to experts show a higher activation (i.e., operationalized by HbR) in two widespread clusters that compromise the following cortical regions: (i) Cluster 1 in the left, contralateral hemisphere including the SMA, PMC, M1, and S1 as well as (i) Cluster 2 in the right, ipsilateral hemisphere including the M1, S1, IPC, SPC and STG.

While previous studies (Balardin et al. 2017; Carius et al. 2021) also observed a more pronounced activation of specific brain areas such as the PMC, M1, and IPC during the execution of table tennis strokes, a higher activation in those areas in table tennis experts as compared to novices was noticed (Carius et al. 2021). This contrasting finding seems to be related to differences in study methodology. In our previous study, we did not control for movement frequency to preserve the ecological validity of the task (Carius et al. 2021). However, given the fact (i) that movement frequency can alter cortical activity patterns in prefrontal and motor-related brain areas (Guérin et al. 2021, 2022; Kuboyama et al. 2004, 2005), and (ii) that table tennis experts in our previous study performed significantly more strokes than novices (i.e., exhibited a higher movement frequency), the higher activation observed in the previous study in table tennis experts is probably related to the higher movement frequency rather than reflecting expertise-related differences in cortical activity (Carius et al. 2021).

The expertise-related effects being observed in the current study (i.e., higher activation in table tennis novices as compared to experts), in which we apply a more rigorous study design (i.e., controlled for movement frequency), fit to the neural efficiency hypothesis postulating that experts utilize cortical resources more efficiently than novices (e.g., higher activation on task-relevant and “compensational” brain regions in novices as compared to experts) (Li & Smith 2021; Neubauer & Fink 2009). In line with the neural efficiency hypothesis, the lower activation of motor-related areas such as the PMC and M1 in table tennis experts probably mirror a more automatized planning, preparation, and execution of table tennis strokes as such functions are generally attributed to these brain areas (Leff et al. 2011). The higher activation of the inferior partial cortex—namely the angular gyrus—in table tennis novices in comparison to experts might reflect a higher reliance of novices on multisensory and sensorimotor integration (i.e., lower neural efficiency) to perform table tennis strokes as the inferior parietal cortex in general (Bruner 2018; Culham & Valyear 2006; Fogassi & Luppino 2005; Freedman & Ibos 2018) and the angular cortex in particular (Seghier 2013) play an important role in different processes of multisensory and sensorimotor integration (e.g., in visuomotor actions such as grasping, reaching and eye-movements).

Concerning prefrontal cortex activation, there is evidence that table tennis novices as compared to experts exhibit a higher activation of prefrontal areas (e.g., middle frontal gyrus) during sport-related and -unrelated tasks (Guo et al. 2017). Such an activation of prefrontal structures during specific task is interpreted as a typical sign of a more controlled (“compensational”) and less automatized (motor) control (Herold et al. 2017a, b). However, in the current study, we are not able to draw solid conclusion with regard to expertise-related differences in prefrontal cortex activation since no clear pattern in concentration changes of the chromophores (neither in HbO nor in HbR) emerged. In particular, we did observe higher concentration changes of HbO in dlPFC in novices as compared to experts, but did not identify such a difference for HbR. As a higher brain activation is typically mirrored in a task-related increase in the concentration of HbO and a concomitant decrease of HbR (Herold et al. 2018; Scholkmann et al. 2022), a sole increase in HbO cannot be reliably interpreted as an indicator for a higher brain activation of table tennis novices, which, in turn, somewhat limits the interpretation of such a finding.

#### Task Effects

In addition to expertise-related differences, we also observed task-based differences in cortical activity. More specifically, task-related effects were observed in a widespread cluster comprising of bilateral dlPFC, MFG, SMA, PMC, M1, MTG, STG, SPC and IPC as well as S1_L_ (see result section for a more detailed overview). Here, the current study has addressed the majority of the limitations of our previous study such as the standardization of the movement frequency and the utilization of a randomized sequence of strokes (i.e., RD) instead of a fixed one. Especially, the latter point, the transition from FH or BH to RD, is probably a greater challenge for the motor control of novice and expert table tennis players than the transition from FH to FHBH—as applied in our previous study (Carius et al. 2021). However, our findings that cortical activity vary as a function of task complexity are consistent with the observations of previous studies investigating the influence of motor complexity on cortical activity using tasks such as finger tapping (Holper et al. 2009), juggling (Carius et al. 2016) or table tennis strokes (Balardin et al. 2017). For instance, in the single-subject study of Balardin et al. (2017) in which cortical activity of one expert table tennis player were studied during different table tennis strokes (i.e., FH and BH), higher PMC activity in unpredictable (i.e., unpredictable strokes to FH or BH) in comparison to predictable (i.e., strokes to FH) table tennis strokes was observed (Balardin et al. 2017). Given the observation of other studies showing that the PMC plays a crucial role in movement planning and monitoring (Leff et al. 2011; Pearce & Moran 2012; Pesaran et al. 2006), our findings suggest that different types of table tennis strokes might rely on higher level movement planning and monitoring which is mirrored by an increased activation of the PMC. This line of interpretation is supported by recent evidence showing that higher task complexity is associated with more pronounced activation of PMC (Maes et al. 2020; Meister et al. 2005; Swinnen & Wenderoth 2004). Comparably, our results of dlPFC activation in more complex task conditions are consistent with the findings that frontal areas (e.g., dlPFC) are activated (i) when a (motor) task becomes more complex (Serrien et al. 2007), and (ii) when motor control is less automatized and is controlled by the indirect locomotor pathway (Herold et al. 2017a, b).

Regarding post hoc comparisons of the different task conditions (i.e., FH, BH, and RD), FH and BH showed various differences depending on the brain area. In particular, a higher concentration of HbR in bilateral M1, S1_L_, and SPC_L_ was observed during the execution of FH, whereas we noticed a higher concentration of HbR in bilateral MTG, STG, and IFGr during the execution of BH. These findings suggest that FH poses higher demands on sensorimotor integration, while BH requires a higher level of multisensory integration, although more research is necessary to further corroborate the evidence for these assumptions.

Based on the findings (i) that HbR concentrations in bilateral MFG, SMA_L,_ and SPC_L_ are higher during RD as compared to BH, and (ii) that HbR concentrations are higher in bilateral MTG, STG, and IFG during RD as compared to FH, it seems reasonable to assume that, from a neurobiological point of view, RD is the condition with the highest task complexity (i.e., as compared to FH and BH). Such a higher task complexity might be related to switch costs that occur in RD requiring participants to switch in a random and unpredictable manner between two different table tennis stroke techniques (i.e., FH and BH) as compared to FH and BH in which the participants executed only one table tennis stroke technique. This line of interpretation is supported by other neuroimaging studies that observed that switch costs (i.e., in a cognitive task) were associated with a more pronounced activation of brain areas being located in premotor cortex (e.g., SMA, Cutini et al. 2008; Dove et al. 2000) and/or parietal cortex (Kimberg et al. 2000; Petruo & Beste 2021) which resembles the observations of the current study. In addition, the assumption of the highest task complexity of RD is at least partly supported by our finding showing that novices have a lower target accuracy in the RD condition in comparison to FH and BH.

The higher movement speeds of FH and RD compared with BH may also account for the higher concentration changes of HbR in bilateral M1, S1_L_, and SPC_L_ during execution of FH compared with BH and bilateral MFG, SMA_L_, and SPC_L_ during execution of RD compared with BH, respectively (see Results section for Behavioral Data). Although we standardized movement frequency, we did not standardize movement speed. This limitation restricts the results with respect to task-related differences in cortical brain processing. However, a generalized higher concentration change of HbR as a consequence of larger movement speed could not be observed. This finding makes it rather unlikely that movement speed per se is the main driver of our current findings.

Whether these task-related differences in cortical activity can be used to monitor a training and/or to adjust specific exercise variables seems to be a promising area for further research (Herold et al. 2020; Stéphane Perrey, 2022; Seidel-Marzi & Ragert 2020).

## Limitations

The current study addressed the majority of the limitations of our previous study (Carius et al. 2021) such as the standardization of movement frequency, ball placement via a robot, and assessment of whole-head cortical activity, but some points should be considered when interpreting our findings.

Firstly, although we observed that the ratings of some psychological confounders (i.e., level of discomfort) slightly increased from pre- to post-test, we did not notice significant changes in systemic physiological confounders (i.e., mean heart rate). Thus, it seems unlikely that such changes have seriously influenced our results and fNIRS data quality, although it has to be acknowledged that the activation patterns observed in the temporal lobe—namely differences in MTG and STG—should be interpreted cautiously as there is evidence in the literature that the temporal muscle of the head can confound the fNIRS signal to some extent (Schecklmann et al. 2017). Research furthermore suggests, that the temporal lobe represents a critical hub in the regulation of autonomic cardiovascular function (Dono et al. 2020), which might relate to the execution of sports-specific movements in our study. However, as we followed recent recommendations concerning the processing of fNIRS data (Scholkmann et al. 2022; Tachtsidis & Scholkmann 2016; Yücel et al. 2021) and applied state-of-the-art techniques (i.e., data quality check via scalp coupling index, short-separation channels regression) to ensure high data quality, the influence of potential confounders (i.e., systemic physiological changes) is probably low.

Secondly, as fNIRS neuroimaging allows to cover hemodynamic alterations only in cortical layers, it is not possible to provide information whether expertise level or task complexity modulates the activation of subcortical structures as well since such structures are highly relevant for the control of sport-specific movements and influenced by the level of motor expertise (e.g., cerebellum, basal ganglia) (Park et al. 2009; Roberts et al. 2013; Taubert et al. 2015; Yang 2015; Yarrow et al. 2009; Zhang et al. 2021).

Thirdly, as the current investigation is among the first studies that applied TFCE as a specific method of cluster-based permutation testing in the context of fNIRS neuroimaging, there are some limitations of TFCE that needs to be acknowledged. In particular, TCFE is well-situated to address the multiple comparison problem and to identify a significant differences between conditions, but it does not allow to assess of the statistical significance regarding a specific timepoint (i.e., temporal onset) or precise spatial estimation (i.e., a single optode) (Sassenhagen & Draschkow 2019). Thus, the interpretation of TCFE results can be somewhat challenging and prone to misinterpretation, if not conducted appropriately. To avoid such pitfalls, we follow recent recommendations on how to report the results of the TCFE analysis (Sassenhagen & Draschkow 2019).

## Conclusion

In summary, our results suggest that there are expertise-related differences between table tennis experts and novices in widespread clusters compromising sensorimotor and multisensory brain areas, whereas novices exhibit, in general, a higher activation in those areas as compared to experts. The latter finding provides further empirical evidence for the neural efficiency hypothesis which postulates that experts can solve a specific task with lower neural resources (i.e., mirrored in lower activation of specific brain areas). Furthermore, we observed task-specific differences in cortical activity concerning FH, BH, and RD suggesting that the task complexity is probably reflected in distinct brain activation patterns. Whether our findings can be useful to monitor and tailor sport-specific training interventions requires future investigations.

## Supplementary Information

Below is the link to the electronic supplementary material.

Supplementary file1 (DOCX 1478 KB)

Supplementary file2 (DOCX 19 KB)

## Data Availability

All data that support the findings of this study are available from the corresponding author, D.C. if a formal data sharing agreement exists. Besides, all software used in the present study is open-source and as such publicly available.
