# Supplementary material for: Increased Cortical Activity in Novices Compared to Experts During Table Tennis: A Whole-Brain fNIRS Study Using Threshold-Free Cluster Enhancement Analysis
Source: Brain Topogr. 2023 Apr 29;36(4):500–16. doi: 10.1007/s10548-023-00963-y (PMC10293405; doi:10.1007/s10548-023-00963-y)
Supplement: Supplementary file 1 — Supplementary file1 (DOCX 1478 KB) [file 10548_2023_963_MOESM1_ESM.docx]

| 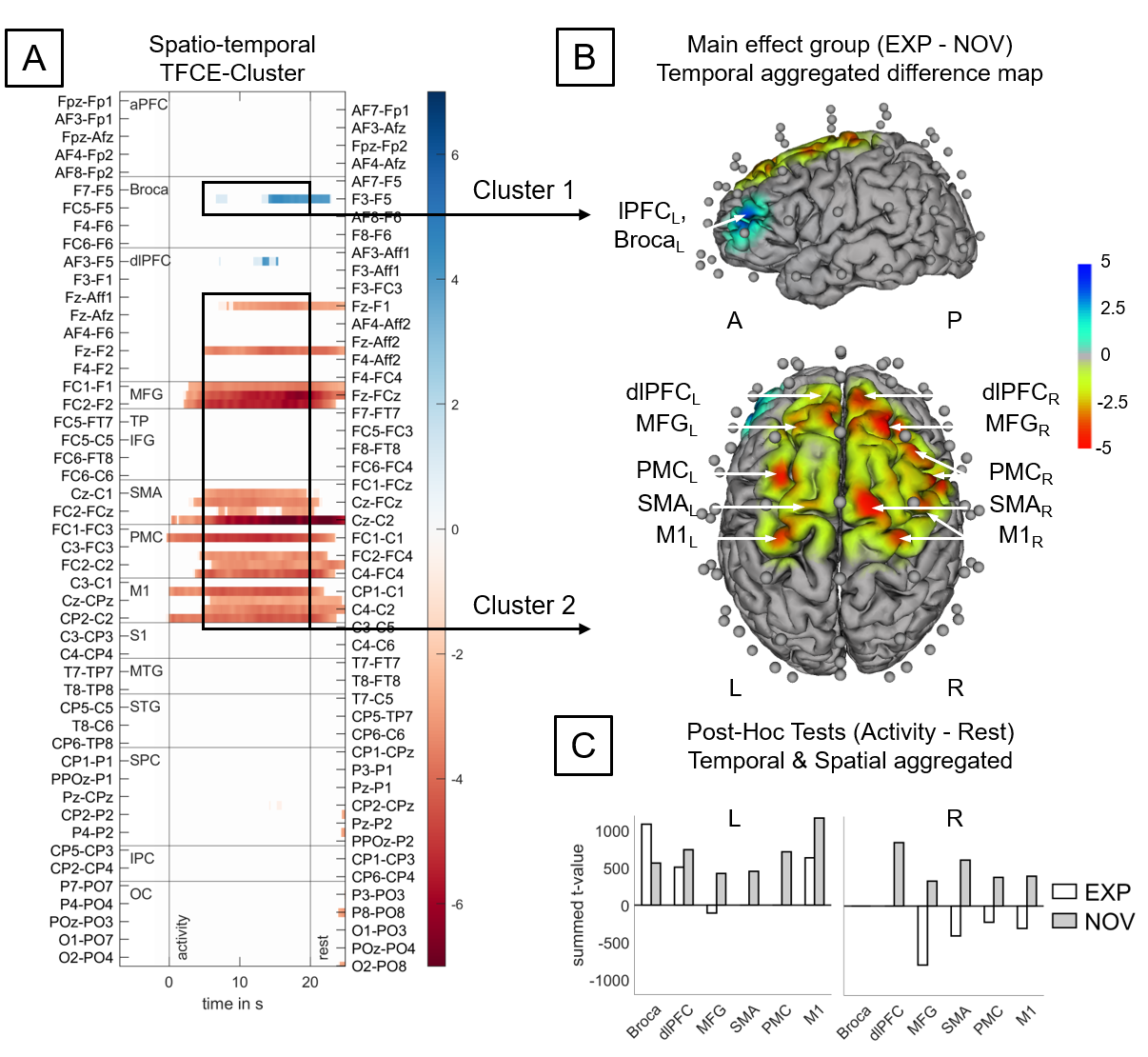 |
| --- |
| **Supplementary fig. 1 Group effects on oxygenated hemoglobin (HbO) concentration changes during table tennis** (Novice [NOV] vs. Experts [EXP] executing forehand and backhand strokes) according to TFCE analysis. A Raster diagram showing significant data points (spatio-temporal cluster). Rectangles indicate channel/time points modulated by expertise. Blue rectangles indicate higher concentration changes for experts. Red rectangles indicate higher concentration changes for novices. The colorbar indicates TFCE t-values (post-Hoc Test Expertise). Note that channels (source-detector combinations) are organized along the y-axis according to their associated brain areas (Zimeo Morais, Balardin, & Sato, 2018). B Temporal aggregated difference map (EXP - NOV). Optodes (transmitters and detectors) are shown for the topographic images; colors represent mean TFCE t-values (sample range 5-20 sec). Images are thresholded at p < 0.05. Upper illustration (left view): Cluster 1: Channels indicate higher concentration changes for experts (two channel including left dlPFC and left Broca, timerange 12-23 sec, *t*(20)_max_ = 4.43, *p*_max_ = .039). Illustration below (top view): Cluster 2: All channels indicate higher concentration changes for novices (54 channel including bilateral dlPFC, FEF, SMA, PMC, M1, timerange 0-40 sec, *t*(20)_max_ = -7.47, *p*_max <_ .001). C Post-hoc tests activity vs. baseline (rest) concentration changes. Bars represent novices resp. experts. Values are summed TFCE t-values (Cluster 1 & 2, sample range 5-20 sec). for all significant channel belonging to the aforementioned cluster resp. brain areas. Abbreviations: TFCE, threshold-free cluster enhancement, L: left hemisphere, R: right hemisphere |

| 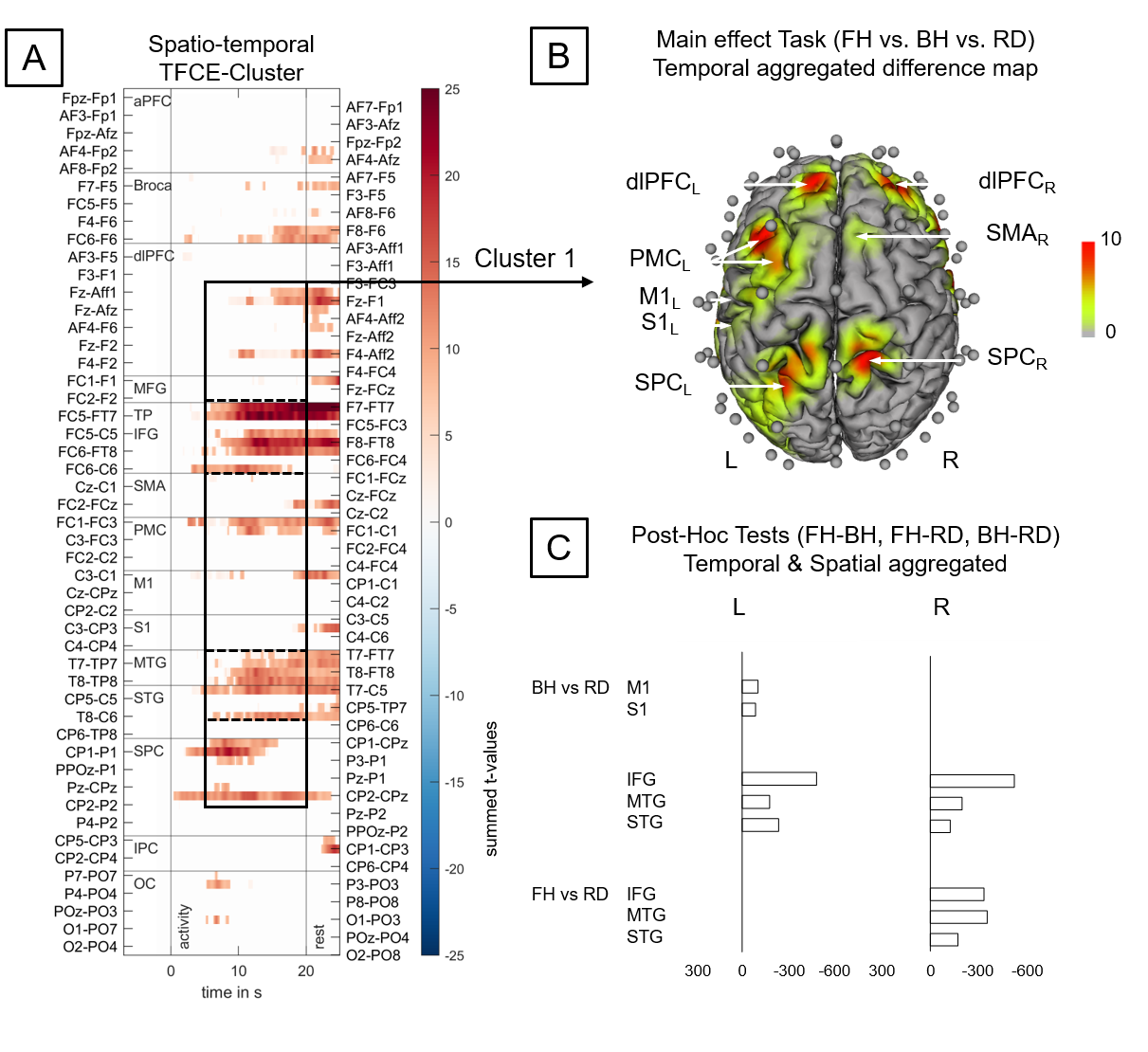 |
| --- |
| **Supplementary fig. 2 Task effects on oxygenated hemoglobin (HbO) concentration changes during table tennis** (Forehand vs. backhand vs. Forehand/backhand strokes) according to TFCE analysis. A Raster diagram showing significant data points (spatio-temporal cluster). Rectangles indicate channel/time points modulated by task. The colorbar indicates TFCE f-values (post-Hoc Test Task). Note that channels (source-detector combinations) are organized along the y-axis according to their associated brain areas (Zimeo Morais, Balardin, & Sato, 2018). B Optodes (transmitters and detectors) are shown for the topographic images; colors represent mean TFCE f-values (sample range 5-20 sec). Images are thresholded at p < 0.05 (Top view, Cluster 1: 66 channel including bilateral aPFC, Broca, dlPFC, SMA, MTG, STG, SAC and FEF_L_, PMC_L_, M1_L_, S1_L_, SMG_L_ and An39_L_, timerange 0-40 sec, *F*(2,42)_max_ = 28.20, *p*_max_ <.001). C Bars represent post-hoc comparisons between FH, BH & RD. Values are summed TFCE t-values (sample range 5-20 sec). Abbreviations: TFCE, threshold-free cluster enhancement, L: left hemisphere, R: right hemisphere, SMA: supplementary motor cortex, PMC: premotor cortex, M1: primary motor cortex, S1: primary somatosensory cortex, SAC: somatosensory association cortex, SMG: gyrus supramarginalis |
