# Supplementary material for: Increased Cortical Activity in Novices Compared to Experts During Table Tennis: A Whole-Brain fNIRS Study Using Threshold-Free Cluster Enhancement Analysis
Source: Brain Topogr. 2023 Apr 29;36(4):500–16. doi: 10.1007/s10548-023-00963-y (PMC10293405; doi:10.1007/s10548-023-00963-y)
Supplement: Supplementary file 2 — Supplementary file2 (DOCX 19 KB) [file 10548_2023_963_MOESM2_ESM.docx]

| Supplementary Table 1: Group and task effects on target accuracy during table tennis. Repeated measures ANOVA results for total sample (*n* = 33) & fNIRS subsample (*n* = 20). | | | | | | |
| --- | --- | --- | --- | --- | --- | --- |
|  | *SS* | *df* | *MS* | *F* | *P* | *η_p_^2^* |
| Total sample (n = 33) |  |  |  |  |  |  |
| Task | 2449 | 1.52 | 1609 | 7.39 | .004 | .19 |
| Task x Group | 1130 | 1.52 | 743 | 3.41 | .054 | .10 |
| Residuals | 10279 | 47.16 | 217 |  |  |  |
| Group | 17076 | 1.00 | 17076 | 15.36 | < .001 | .33 |
| Residuals | 34463 | 31.00 | 1111 |  |  |  |
| Subsample (n = 20) |  |  |  |  |  |  |
| Task | 1299 | 1.46 | 892 | 3.85 | .046 | .18 |
| Task x Group | 847 | 1.46 | 581 | 3.41 | .114 | .12 |
| Residuals | 6082 | 26.23 | 231 |  |  |  |
| Group | 6984 | 1.00 | 6984 | 5.20 | .035 | .22 |
| Residuals | 24175 | 18.00 | 1343 |  |  |  |
| Note. Target accuracy from two novices could not be evaluated due to camera recording errors (total sample: *n* = 33, 17 experts & 16 novices; subsample: *n* = 20, 11 experts & 9 novices) | | | | | | |

| Supplementary Table 2: Group differences regarding attention, fatigue and discomfort before and after the examination. Results for fNIRS subsample (*n* = 22). | | | | | | |
| --- | --- | --- | --- | --- | --- | --- |
|  | *Experts* |  | *Novices* |  |  |  |
|  | *Mean* | *SD* | *Mean* | *SD* | *U* | *p* |
| *Pretest* |  |  |  |  |  |  |
| Attention | 8.0 | 1.0 | 7.0 | 1.0 | 89.0 | .055 |
| Fatigue | 8.0 | 1.0 | 8.0 | 1.0 | 66.5 | .710 |
| Discomfort | 1.0 | 0.0 | 1.0 | 0.0 | 55.5 | .687 |
| *Posttest* |  |  |  |  |  |  |
| Attention | 8.0 | 1.0 | 8.0 | 1.0 | 51.5 | .558 |
| Fatigue | 7.0 | 1.0 | 8.0 | 1.0 | 43.5 | .257 |
| Discomfort | 2.0 | 1.0 | 2.0 | 1.0 | 68.5 | .608 |
| *Pretest - Posttest* |  |  |  |  |  |  |
| Attention | -1.0 | 1.0 | 1.0 | 1.0 | 29.0 | .037 |
| Fatigue | 0.0 | 1.0 | 0.0 | 1.0 | 43.0 | .244 |
| Discomfort | 1.0 | 1.0 | 1.0 | 1.0 | 68.5 | .606 |

| Supplementary Table 3: Group and task effects on heart rate during table tennis. Repeated measures ANOVA results for total sample (*n* = 33) and fNIRS subsample (*n* = 20). | | | | | | |
| --- | --- | --- | --- | --- | --- | --- |
|  | *SS* | *df* | *MS* | *F* | *p* | *η_p_^2^* |
| Total sample (n = 33) |  |  |  |  |  |  |
| Task | 13 | 1.45 | 9 | 1.51 | .232 | .06 |
| Task x Group | 17 | 1.45 | 12 | 1.97 | .162 | .06 |
| Residuals | 270 | 45.02 | 6 |  |  |  |
| Group | 528 | 1.00 | 528 | 1.37 | .251 | .04 |
| Residuals | 11976 | 31.00 | 386 |  |  |  |
| Subsample (n = 20) |  |  |  |  |  |  |
| Task | 21 | 1,32 | 16 | 2.14 | .152 | .11 |
| Task x Group | 20 | 1.32 | 15 | 2.07 | .160 | .10 |
| Residuals | 173 | 23.80 | 7 |  |  |  |
| Group | 215 | 1.00 | 215 | 0.54 | .472 | .03 |
| Residuals |  |  |  |  |  |  |
| Note. Heart rate from two novices could not be evaluated due to recording errors (total sample: *n* = 33, 17 experts & 16 novices; subsample: *n* = 20, 11 experts & 9 novices) | | | | | | |
